# Role of the supine lateral radiograph of the spine in vertebroplasty for osteoporotic vertebral compression fracture: a prospective study

**DOI:** 10.1186/1471-2474-11-164

**Published:** 2010-07-19

**Authors:** Meng-Huang Wu, Tsung-Jen Huang, Chin-Chang Cheng, Yen-Yao Li, Robert Wen-Wei Hsu

**Affiliations:** 1 Department of Orthopedic Surgery, Chang Gung Memorial Hospital at Chiayi, Chiayi, Taiwan; 2 College of Medicine, Chang Gung University, Taoyuan, Taiwan; 3 Department of Orthopedic Surgery, Xiamen Chang Gung Hospital, Xiamen, Fujian, China

## Abstract

**Background:**

Severely collapsed vertebral compression fracture (VCF) is usually considered as a contraindication for vertebroplasty because of critically decreased vertebral height (less than one-third the original height). However, osteoporotic VCF can possess dynamic mobility with intravertebral cleft (IVC), which can be demonstrated on supine lateral radiographs (SuLR) and standing lateral radiographs (StLR). The purposes of this study were to: (1) evaluate the efficacy of SuLR to detect IVCs and assess the intravertebral mobility in VCFs, and (2) evaluate the short-term results of vertebroplasty in severely collapsed VCFs with IVCs.

**Methods:**

We enrolled 37 patients with 40 symptomatic osteoporotic VCFs for vertebroplasty; 11 had severely collapsed VCFs with concurrent IVCs detected on the SuLR, the others had not-severely collapsed VCFs. A preoperative StLR, SuLR, magnetic resonance imaging (MRI), and postoperative StLR were taken from all patients. Radiographs were digitized to calculate vertebral body morphometrics including vertebral height ratio and Cobb's kyphotic angle. The intensity of the patient's pain was assessed by the visual analogue scale (VAS) on the day before operation and 1 day, 1 month, and 4 months after operation. The patient's VAS scores and image measurement results were assessed with the paired *t*-test and Pearson correlation tests; Mann-Whitney U test was used for VAS subgroup comparison. Significance was defined as *p *< 0.05.

**Results:**

IVCs in patients with not-severely collapsed VCFs were detected in 21 vertebrae (72.4%) by MRI, in 15 vertebrae (51.7%) by preoperative SuLR, and in 7 vertebrae (24.1%) by preoperative StLR. Using the MRI as a gold standard to detect IVCs, SuLR exhibit a sensitivity of 0.71 as compared to StLR that yield a sensitivity of 0.33. In patients with VCFs with IVCs detected on SuLR, the average of the postoperative restoration in vertebral height ratio was significantly higher than that in those without IVCs (17.1% vs. 6.4%). There was no statistical difference in the VAS score between severely collapsed VCFs with IVCs detected on SuLR and not-severely collapsed VCFs at any follow-up time point.

**Conclusions:**

The SuLR efficiently detects an IVC in VCF, which indicates a better vertebral height correction after vertebroplasty compared to VCF without IVC. Before performing a costly MRI, SuLR can identify more IVCs than StLR in patients with severely collapsed VCFs, whom may become the candidates for vertebroplasty.

## Background

Vertebroplasty is a minimally invasive surgical procedure that can relieve pain caused by an osteoporotic vertebral compression fracture (VCF) [1-4]. However, VCFs that show severe vertebral body collapse to less than one-third of its original height were considered as a contraindication for vertebroplasty, because the vertebral height (VH) was too low which might hinder needle placement within the vertebral body [5,6]. Furthermore, severely collapsed VCFs are often accompanied by significant kyphosis associated with an inferior outcome after vertebroplasty [2]. However, we had observed that the VH of severely collapsed VCFs increased on supine lateral radiographs (SuLR) compared with standing lateral radiographs (StLR). McKiernan et al. [7] had reported that SuLR could demonstrate the dynamic mobility of the vertebral body and formation of intravertebral clefts (IVCs) in nonunion of VCFs. These mobile VCFs are mechanically unstable and can be managed by vertebroplasty or kyphoplasty [8]. Significant height recovery and improved sagittal alignment after postural reduction could be demonstrated for these mobile, severely collapsed VCFs resulting in pain relief after vertebroplasty or kyphoplasty [8,9]. The purposes of this study were to: (1) evaluate the efficacy of SuLR to detect IVCs and assess the intravertebral mobility in osteoporotic VCFs in comparison with StLR or magnetic resonance imaging (MRI) and (2) evaluate the short-term results of vertebroplasty in severely collapsed VCFs with IVCs detected on SuLR.

## Methods

### Subjects

This prospective study was approved by the institutional review board of our hospital (No 99-0445B). Thirty-seven patients (29 females and 8 males; mean age, 75 years; age, 50-93 years; 40 vertebrae) with symptomatic osteoporotic VCFs were enrolled from July 2008 to April 2009 according to the CONSORT statement [10]. All of them had given informed consent to participate the study and underwent percutaneous vertebroplasty with cement augmentation and were followed up for at least 6 months. The indication for percutaneous vertebroplasty was painful osteoporotic VCF refractory to conservative treatment with severe local tenderness over the spinal process of the fractured vertebra and without focal radicular pain. Besides, in patients with severely collapsed VCFs with residual VH less than 33% of the original height, IVCs should be noted on SuLR before considering the patient for percutaneous vertebroplasty.

### Radiographic outcome measures

**Figure 1:**
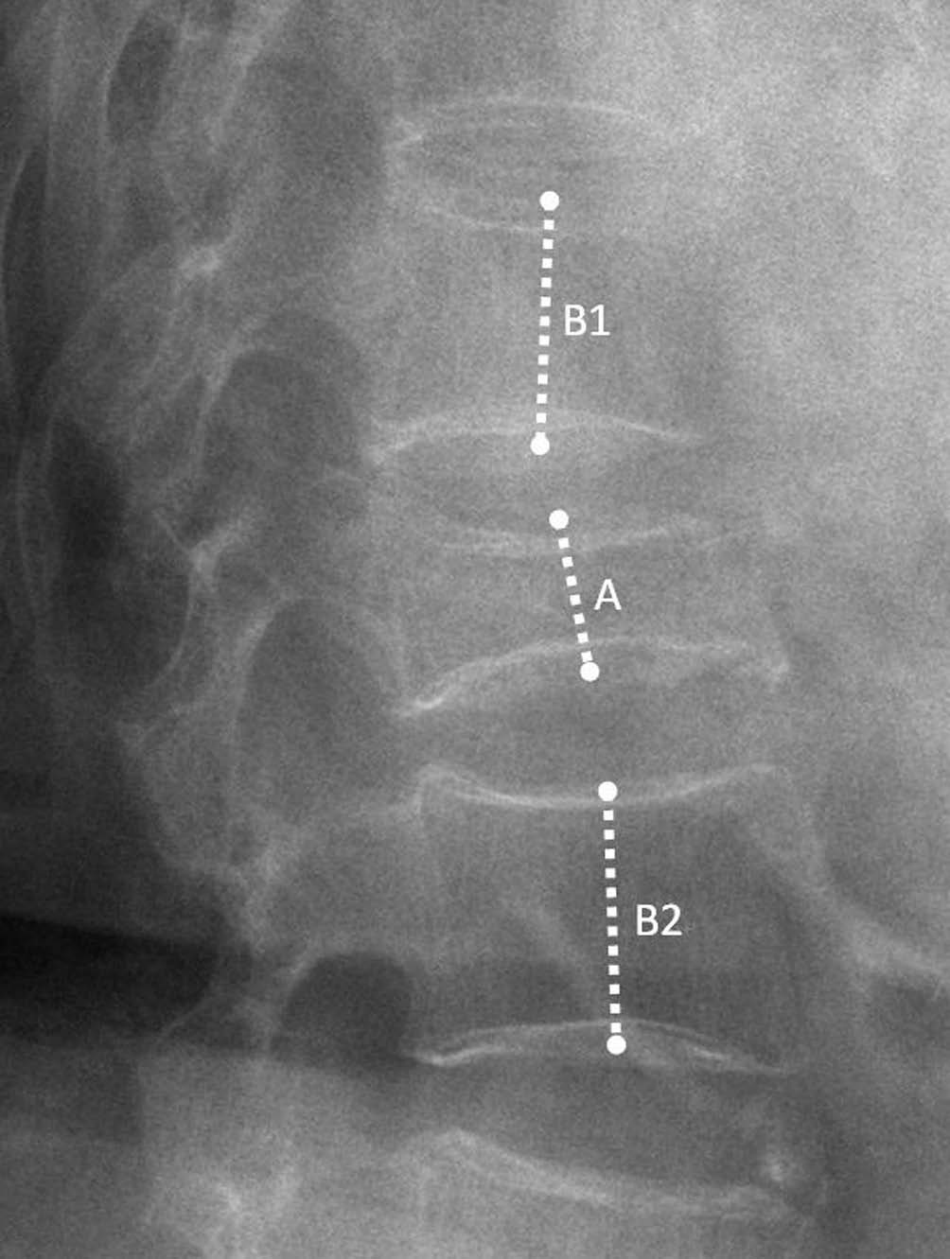
**The measurement of vertebral height ratio**. The vertebral height (VH) is the distance between the midpoints of upper and lower endplates of the index vertebra on the lateral views. The VH ratio of the index vertebra was then calculated as the VH of the index vertebra (A) divided by the average of the VHs 1 level above (B1) and below the index vertebra (B2).

Within 1 week before operation, standing anteroposterior (AP) radiograph, StLR, and SuLR of the thoracic-lumbar spine were taken from all patients to identify the fractured vertebra and determine the mobile vertebra with or without IVC. MRIs were also obtained from all patients on the day of or 1 day before operation to exclude infection or pathologic fracture and to identify IVC [11]. On the day after operation, postoperative StLR was taken to evaluate the restoration of the VH and regional kyphosis, compared with preoperative StLR. The 3 radiographs (preoperative StLR, SuLR, and postoperative StLR) were digitized to measure the VH of the index vertebra, which was the distance between the midpoints of upper and lower endplates on the lateral views described by McKiernan et al. [7] (Figure 1). The VH ratio of the index vertebra was then calculated as the VH of the index vertebra divided by the average of the VH 1 level above and below the index vertebra. The regional kyphotic angles were also calculated with Cobb's method to measure the angle formed between a line drawn parallel to the superior endplate of 1 vertebra above the index vertebra and a line drawn parallel to the inferior endplate of the vertebra 1 level below the index vertebra [12]. Postoperative radiographs were also used to detect cement leakage.

### Clinical outcome measures

The intensity of the patient's pain was assessed by the visual analog scale [13] (VAS, 0 to 10, 0 representing no pain and 10 representing worst pain ever experienced) at rest on the day before the procedure. The pain was reassessed on the first day after operation, and 1 month and 4 months postoperatively. Eight patients were excluded from the VAS score analysis due to postoperative infection (n = 1), previous spinal instrumentation (n = 3), concurrent 2-level operation (n = 3), and subsequent adjacent vertebral compression fracture (n = 1). Therefore, the data of 29 patients were finally used for analysis of the VAS score.

### Statistical methods

All statistical analyses were performed with the Statistical Package for the Social Sciences software (SPSS, version 12.0, SPSS Inc. Chicago, Il). The patient's VAS scores and image measurement results were assessed with the paired t-test and Pearson correlation tests; Mann-Whitney U test was used for VAS subgroup comparison. Significance was defined as *p *< 0.05.

## Results

**Figure 2:**
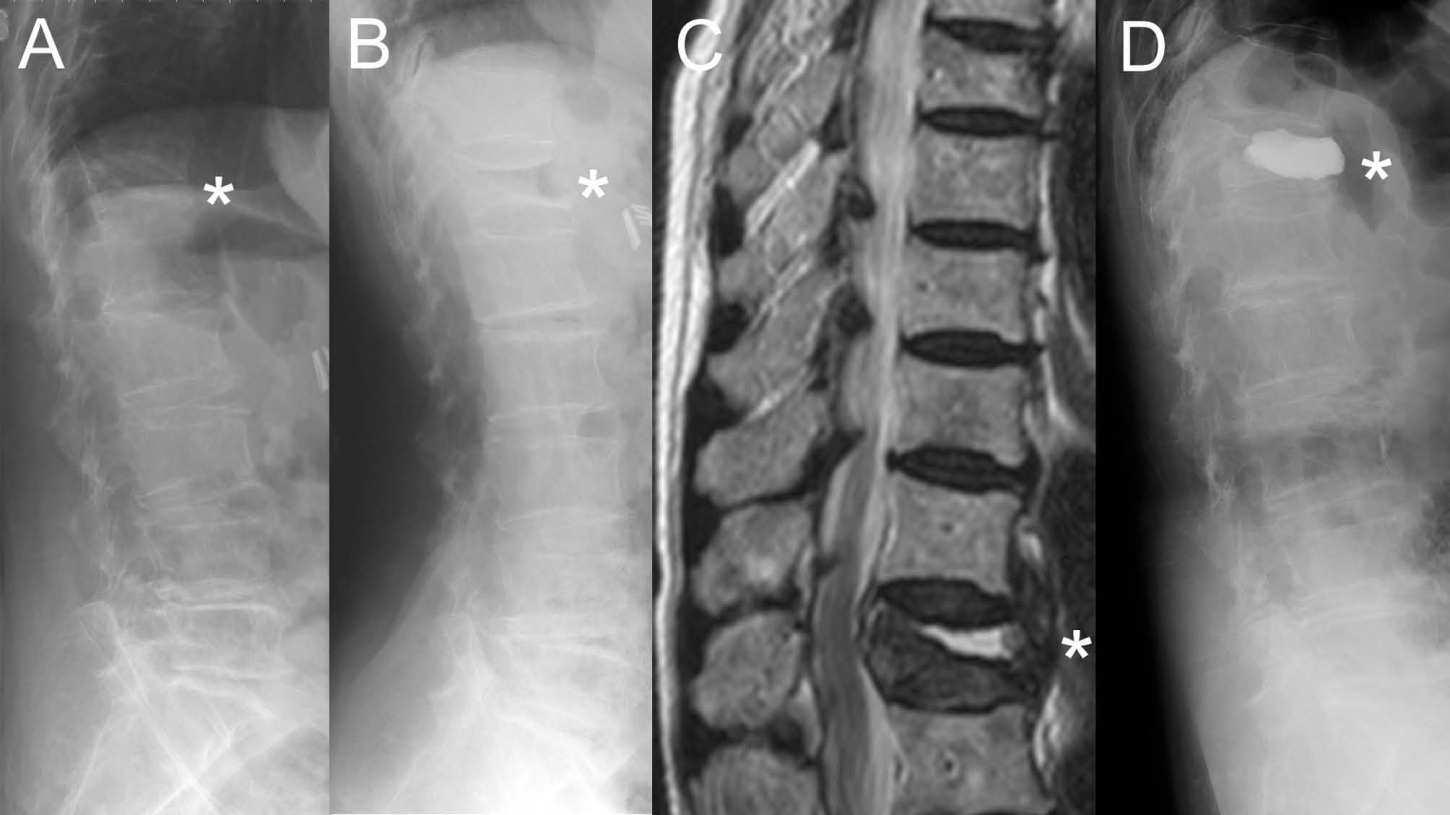
**The intravertebral cleft detected on the supine lateral radiograph**. A 93-year-old male patient with T12 severely collapsed vertebral compression fracture (VCF) with preoperative VAS score of 9, vertebral height (VH) ratio of 24.5%, and a kyphotic angle of 31.9° on the preoperative standing lateral radiograph (StLR) (A). The supine lateral radiograph (SuLR) (B) revealed an intravertebral cleft (IVC) and the VH ratio and kyphotic angle were reduced to 39.9% and 14.1°, respectively. We performed T2-weighted MRI (C) to confirm the level of symptomatic VCF at 12 weeks after a fall from standing height, which showed an IVC with fluid sign. The postoperative StLR (D) showed that the VH ratio and kyphotic angle were restored to 60% and 24.3°, respectively. The VAS score of this patient decreased to 3, 1, and 0 points at day 1, 1 month, and 4 months, respectively, post operation. (* T12 vertebra)

Thirty-seven consecutive patients (40 VCFs) underwent vertebroplasty in our study. The average time between fracture and vertebroplasty was 14.7 weeks (4~144 weeks). The lesions were mostly located at the thoracolumbar junction (T11-L1, 57.5%). Eleven vertebrae were diagnosed as severely collapsed VCF with concurrent IVC detected on the SuLR (Figure 2).

In 29 not-severely collapsed VCFs, IVCs were detected in 21 vertebrae (72.4%) by MRI, in 15 vertebrae (51.7%) on the preoperative SuLR, and in 7 vertebrae (24.1%) on the StLR. Using the MRI as a gold standard to detect IVCs, SuLR exhibit a sensitivity of .71 as compared to StLR that yield a sensitivity of .33. In the 40 VCFs, the average VH ratio on pre-op StLR was 51.6% (14.8%~89.6%), on pre-op SuLR was 56.2% (35.4%~92.1%), and on post-op StLR was 62.3% (40.0%~93.9%). The average Cobb's angle on pre-op StLR was 21.5° (-2°~47.9°), on pre-op SuLR was 15.5° (-10°~47.1°), and on post-op StLR was 17.7° (-6.6°~41.9°).

The correlations between preoperative and postoperative radiographic parameters were evaluated with Pearson correlation test (Table 1). There was a strong positive correlation between the pre-op difference in VH ratio between pre-op StLR and SuLR and the post-op restoration in VH ratio comparing pre-op StLR with post-op StLR (*p *< 0.001). When the difference in the VHR and the changes in Cobb's angle were further evaluated, 2 groups of patients could be identified: (1) those patients in which IVC was detected on the SuLR ("supine IVC group") and (2) those in which IVC was not detected on the SuLR ("no supine IVC group"). Also, the pre-op difference and the post-op restoration in VH ratio in the "supine IVC group" were both statistically higher than that in the "no supine IVC group" (*p *= 0.027 and 0.032, respectively, Table 2). The pre-op difference in Cobb's angle between pre-op StLR and SuLR in the "supine IVC group" was statistically higher than that in the "no supine IVC group" (*p *< 0.001). However, there was no significant difference in post-op change in the Cobb's angle comparing pre-op StLR with post-op StLR between either group.

**Table 1:** Pearson correlation between radiographic parameters

**Preoperative Factors**	**VHR1**	**VHR2**	**A1**	**A2**	**VHR2-VHR1**	**A1-A2**
**Postoperative Factors**						
	
A3	N	(-)	(+)	(+)	N	N
VHR3-VHR1	(-)	N	N	N	(+) < 0.001	N
A1-A3	N	N	N	N	N	(-)

VHR: vertebral height ratio, VHR1: Vertebral height ratio on the pre-op standing lateral view, VHR2: Vertebral height ratio on the pre-op supine lateral view, VHR3: Vertebral height ratio on the postoperative standing lateral view, A1: Cobb's kyphotic angles on the pre-op standing lateral view, A2: Cobb's kyphotic angles on the pre-op supine lateral view, A3: Cobb's kyphotic angles on the post-op standing lateral view

-: Negative correlation with *p *< 0.05

+: Positive correlation with *p *< 0.05

N: *p *> 0.05

**Table 2:** IVC on the supine lateral view

	No IVC (N = 14)	IVC (N = 26*)	*p *value
VHR2-VHR1	0.06%	11.3%	0.027
VHR3-VHR1	6.4%	17.1%	0.032
A1-A2	1.1°	9.2°	< 0.001
A1-A3	1.9°	5.6°	0.135

*26 = 11 severely collapsed VCFs + 15 not-severely collapsed VCFs with supine IVCs

IVC: intravertebral cleft, VHR1: Vertebral height ratio on the pre-op standing lateral view, VHR2: Vertebral height ratio on the pre-op supine lateral view, VHR3: Vertebral height ratio on the postoperative standing lateral view, A1: Cobb's kyphotic angles on the pre-op standing lateral view, A2: Cobb's kyphotic angles on the pre-op supine lateral view, A3: Cobb's kyphotic angles on the post-op standing lateral view

The average VAS scores of 29 patients 1 day before operation, and 1 day, 1 month, and 4 months post operation were 8.34, 3.75, 2.88, and 1.84, respectively. The average preoperative VAS score of 9 patients with severely collapsed VCF with supine IVC 1 day before operation, and 1 day, 1 month, and 4 months post operation were 8.56, 3.89, 2.67, and 2.22, respectively. Two patients were excluded due to subsequent adjacent VCF and previous spinal instrumentation, respectively. The average preoperative VAS score of 20 patients with not-severely collapsed VCFs 1 day before operation, and 1 day, 1 month, and 4 months post operation were 8.26, 3.70, 2.96, and 1.70, respectively. There was no statistical difference in the VAS scores between patients with severely collapsed VCFs with supine IVCs and patients with not-severely collapsed VCFs.

### Complications

Cement leakage was noted in 20 of 40 vertebrae (50%). It was found in the disc space of 10 vertebrae, in the paravertebral area of 9 vertebrae, and in the paravertebral soft tissue and vessel of 1 vertebra. We did not note any clinical neurologic deficit due to cement leakage. However, we noted postoperative infection with cement dislodge in 1 vertebra with IVC (L1) 6 months post operation. Thereafter, the patient received anterior corpectomy of L1 and interbody fusion. One patient had a new-onset fracture adjacent to the treated VCF at 1 month post operation and received conservative treatment.

## Discussion

In a VCF, the SuLR can demonstrate dynamic mobility, compared to the standing lateral view [7]. McKiernan et al. defined dynamic mobility of a VCF as any measurable change in VH that occurred between StLR and SuLR. They also found that IVC was present in every mobile fracture. In our study, the detection rate of IVCs in osteoporotic VCFs was higher on SuLR than on StLR. Besides, the VH ratio of SuLR was significantly higher than of StLR. Theoretically, the increased VH seen on SuLR would be also seen by fluoroscopy while the patient is in the prone position and a pillow is placed over the chest and iliac crest during vertebroplasty. Therefore, the increased VH may allow the spinal needle to be easier inserted into the vertebral body. The T2-weighted MRI was superior to both SuLR and StLR regarding IVC detection rate; however, SuLR may be a cost-effective tool before performing a MRI study to select those candidates for vertebroplasty who present with severely collapsed osteoporotic VCFs with IVCs, since SuLR is more than twice as sensitive as StLR in identifying IVC according to our study.

The pre-op difference in VH ratio between StLR and SuLR was highly correlated to the post-op restoration in VH ratio comparing pre-op StLR with post-op StLR. Therefore, the dynamic fracture mobility can be substantial and allows for the possibility of significant VH restoration during the course of vertebroplasty. Since mobile fractured vertebrae can present IVC on SuLR, the likelihood of opening the anterior border for correction during vertebroplasty may be observed [12]. As a result, we consider that the SuLR can efficiently demonstrate an IVC and allows predicting the magnitude of postoperative VH correction, compared with the StLR. This finding supports the theory proposed by McKiernan et al. that VH ratio restoration is likely due to dynamic mobility [14]. They placed the patient in the supine position over a pillow at the level of the index vertebrae to take the lateral radiograph. In our experience, patients in the supine position complained of more pain when a pillow was used than without using it. Furthermore, a high detection rate of IVC has been achieved by MRI, for which the patient lay on the examination table without using a pillow. Therefore, we considered that the supine position alone can make a lateral radiograph more efficient in detecting an IVC compared to the supine position using a pillow. In addition, this would make the patients be more comfortable.

There was no significant difference in the VAS score between the groups with severely collapsed VCFs and that with not-severely collapsed VCFs. In the study of Weill et al. [15], in which 37 patients with metastatic vertebrae underwent 52 vertebroplasty procedures, the lesions were treatable unless the vertebrae had collapsed to less than one-third of the original height. Severely collapsed VCFs had been proposed as a risk factor with a higher complication rate [16]; however, no obvious complication except cement leakage was found in these patients in the study period. Peh et al. reported that 97% of the patients with severe osteoporotic VCFs who underwent vertebroplasty had partial or complete pain relief [6]. Technically, they stated that vertebroplasty in severely collapsed VCFs was not more difficult than in not-severely collapsed VCFs, although they were probably more cautious during the procedure. According to the current study, vertebroplasty can be successfully performed when the severely collapsed VCF harbored an IVC and dynamic mobility, which presented increased VH on the SuLR. We suggest that severely collapsed VCFs with an IVC seen on the SuLR can have good results regarding pain relief compared to not-severely collapsed VCFs once intravertebral stability has been achieved by vertebroplasty.

We further evaluated the relation of the pain score to VH and kyphotic angle. The pre-op VH ratio, post-op VH ratio and their changes did not correlate with the postoperative VAS. Neither did the pre-op Cobb's angle, post-op Cobb's angle and their changes. Though literatures concluded vertebroplasty can restore VH and the kyphotic angle [17-19], there is still no evidence that these factors correlate with pain relief.

### Limitation of study

The manual measuring of the images might have caused an intra-observer and inter-observer error. We used digitized images with a ready-to-use measuring tool, thus simplified the measuring method and reduced the error during measuring. The group of severely collapsed VCFs with IVCs was relatively small to be statistically compared with the group of not-severely collapsed VCFs. Therefore, we used a non-parametric statistic method when we analyzed the data to avoid a statistical error. There were too few patients to draw clinically relevant inferences to the changes in the VAS pain scale. The follow-up time was limited to 6 months, and therefore, this study cannot reflect the effect on mid-term or long-term results.

## Conclusions

The SuLR efficiently detects IVCs in VCFs, which indicates a better VH correction after vertebroplasty compared with VCFs without IVCs. Before performing a costly MRI study, SuLR can be used to identify more IVCs than StLR in patients with severely collapsed VCFs, whom may become the candidates for vertebroplasty.

## Competing interests

The authors declare that they have no competing interests.

## Authors' contributions

MHW participated in the design of the study, analyzed the radiographic measurements and drafted the manuscript. TJH participated in the design of the study and carried out surgeries. CCC participated in the design of the study and assisted in the surgery. YYL conceived the study, carried out surgeries. RWWH participated in the design of the study and coordinated the research groups. All authors read and approved the final manuscript.

## Pre-publication history

The pre-publication history for this paper can be accessed here:

http://www.biomedcentral.com/1471-2474/11/164/prepub
